# Targeted immune activation in pediatric solid tumors: opportunities to complement local control approaches

**DOI:** 10.3389/fimmu.2023.1202169

**Published:** 2023-06-22

**Authors:** Emily P. Vonderhaar, Michael B. Dwinell, Brian T. Craig

**Affiliations:** ^1^ Department of Microbiology and Immunology, Medical College of Wisconsin, Milwaukee, WI, United States; ^2^ Center for Immunology, Medical College of Wisconsin, Milwaukee, WI, United States; ^3^ Cancer Center, Medical College of Wisconsin, Milwaukee, WI, United States; ^4^ Department of Surgery, Medical College of Wisconsin, Milwaukee, WI, United States

**Keywords:** cancer, surgery, radiation, antitumor immunity, pattern-recognition receptor, STING agonist

## Abstract

Surgery or radiation therapy is nearly universally applied for pediatric solid tumors. In many cases, in diverse tumor types, distant metastatic disease is present and evades surgery or radiation. The systemic host response to these local control modalities may lead to a suppression of antitumor immunity, with potential negative impact on the clinical outcomes for patients in this scenario. Emerging evidence suggests that the perioperative immune responses to surgery or radiation can be modulated therapeutically to preserve anti-tumor immunity, with the added benefit of preventing these local control approaches from serving as pro-tumorigenic stimuli. To realize the potential benefit of therapeutic modulation of the systemic response to surgery or radiation on distant disease that evades these modalities, a detailed knowledge of the tumor-specific immunology as well as the immune responses to surgery and radiation is imperative. In this Review we highlight the current understanding of the tumor immune microenvironment for the most common peripheral pediatric solid tumors, the immune responses to surgery and radiation, and current evidence that supports the potential use of immune activating agents in the perioperative window. Finally, we define existing knowledge gaps that limit the current translational potential of modulating perioperative immunity to achieve effective anti-tumor outcomes.

## 1 Introduction

Anti-cancer therapies for solid tumors can be divided into local control approaches (surgery, radiation) and systemic approaches (cytotoxic chemotherapy, targeted biologic agents, immunotherapy). Local control approaches are typically targeted to the primary tumor mass. Surgery is often the preferred approach to primary tumor masses. Radiation is utilized as either primary local control therapy or as an adjuvant approach for incompletely removed tumors, tumors with high risk features, or in organ-preserving surgical approaches such as breast conserving surgery for invasive ductal carcinoma of the breast. Systemic approaches are utilized before local control to increase the efficacy of surgery or radiation by shrinking the primary tumor, or after local control to reduce the risk of metastatic relapse.

Local control is applied differently for pediatric solid tumors in comparison to adult cancers. For adult solid tumors, local control is traditionally only attempted when the disease appears to be localized, without distant metastatic spread at the time of diagnosis. This treatment paradigm is shifting: colon adenocarcinoma metastatic to the liver is now managed with surgical removal of the liver metastases and the primary colon tumor, with dramatic improvements in survival, where previously liver metastases would have relegated the patient to a palliative rather than curative-intent therapeutic strategy (1). In contrast, in many pediatric solid tumors, local control is utilized for primary tumor masses even in situations in which metastatic disease is present at the time of diagnosis (2–4). Metastatic neuroblastoma (NBL) and metastatic Wilms tumor are two examples of clinical scenarios in which major operations for resection of the primary tumor mass is performed in spite of the presence of known distant disease, and this strategy has been employed for several decades. The distant disease is not removed by surgery, and, paradoxically, exposure to surgery can lead to accelerated metastatic progression (5).

Surgery and radiation each produce profound, systemic, off-tumor consequences. Local tissue trauma, anxiety, pain, blood loss, and hypothermia that occur as a part of undergoing an operation combine to produce a “surgical stress” that directly suppresses systemic immune responses (6, 7). On one hand, systemic suppression of the immune system after injury is an adaptive response that serves to restrict immune activation to the site of local tissue injury for tissue repair purposes. However, surgery induced immune suppression may have significant impact on anti-tumor immune responses that can then further complicate and diminish clinical outcomes (8). The systemic effects of radiation are more mixed, in some cases mimicking the suppressive effects of surgery, while in other cases generating or boosting anti-tumor responses (the “abscopal effect”) (9). In the context of cancer, suppressive systemic immune responses evoked by surgery or radiation pose a potential threat to the patient: suppression of antitumor immunity with potential escape and outgrowth of residual tumor cells that were not removed or killed as part of the primary local control procedure. Given the frequency that local control approaches are utilized in pediatric solid tumors despite the presence of metastatic disease, counteracting the suppressive systemic responses to surgery and radiation may offer an opportunity to preserve or augment antitumor immunity and improve outcomes.

Prior to devising strategies that could counteract the potential negative impact of surgery or radiation on antitumor immunity, a comprehensive understanding of the immune phenotypes of pediatric solid tumors is critical. Much is known about tumor-specific immune microenvironments in adult cancer types, however much less is known about the native tumor immune states in pediatric solid tumors. In this Review, we will summarize the current literature on the native immune microenvironment for each of the major pediatric solid tumor types (NBL, Wilms’ tumor, hepatoblastoma, soft tissue sarcomas and bone sarcomas). Systemic responses to both surgery and radiation will be reviewed, as well as how immune activating agents are currently being tested as anticancer therapeutics. We will highlight current knowledge gaps that need to be addressed with pre-clinical investigations prior to testing the potential role for immune activating agents to complement local control approaches in children affected by solid tumors.

## 2 Immune phenotypes of pediatric solid tumors

Immune phenotypes in the tumor microenvironment (TME) have been broadly categorized according to their T cell content and the cancer-immunity cycle (10, 11). “Immune-inflamed” tumors demonstrate T cells within the tumor mass in direct proximity to tumor cells; these tumors rely on inducing a dysfunctional state to prevent tumor cell killing. Melanoma and non-small cell lung cancer are prototypical examples of tumors with this immune phenotype and are most responsive to checkpoint inhibitor therapy. “Immune-excluded” tumors have T cells present, however they are physically separated from tumor cell nests, typically along the perimeter of the tumor, suggesting failure of the T cells to invade the tumor mass despite localizing to the tumor. One possible mechanistic explanation for these “immune-excluded” tumors is an imbalance of immune-activating compared to immune-suppressing signals in the TME. Tolerogenic dendritic cells produce immunosuppressive cytokines such as IL-10 and TGF-β, and demonstrate low expression of peptide complexes and co-stimulatory molecules (12). Finally, “immune-desert” tumors lack T cells at the tumor site, suggesting a failure of migration or antigen recognition.

The presence of tumor-reactive cytotoxic T cells in the TME of immunogenic tumors prompted investigations to identify the underlying pathways driving this natural response to tumor. Immunogenic tumors were associated with a type I interferon (IFN) transcriptional signature, implicating type I IFNs as the effector signaling molecules involved in the generation of spontaneous antitumor T cell responses (13). Type I IFN comprise IFN-α and IFN-β, both of which signal through the ubiquitously expressed type I IFN receptor (IFNAR), forming heterodimers on the cell surface and activating signaling cascades that culminate in the upregulation of a variety of gene products (14, 15). Type I IFNs have been demonstrated to possess several antitumor properties: type I IFN activates dendritic cells by promoting maturation and the cross-priming response (16, 17), enhances survival of activated lymphocytes cells (18), augments clonal expansion and effector differentiation of CD8^+^ T cells *via* cell-intrinsic IFNAR signaling (19), and induces IL-15 to promote the development of memory CD8^+^ T cells (20). Moreover, IFNAR signaling is important in the rejection of highly immunogenic and unedited tumors (21, 22), and in some experimental contexts is required for the generation of antitumor immune responses. Hence, type I IFN signaling promotes progression through the “cancer-immunity cycle” by augmenting cross-presentation by antigen-presenting cells and their migration to lymph nodes, and supporting memory cytotoxic T lymphocyte survival (23). Several immune signaling pathways culminate in type I IFN production. Cytosolic DNA is a potent trigger of the innate immune system through key sensors including Toll-like receptor 9 (TLR9), absent in melanoma 2 (AIM2), DNA-dependent activator of IFN regulatory factors (DAI), and Stimulator of Interferon Genes (STING) that mediate antimicrobial immunity (24). Of these known intracellular DNA sensors, STING was identified as a necessary upstream mediator in the type I IFN-dependent generation of endogenous antitumor immunity in immunogenic tumor types, identifying the STING pathway as a critical bridge to activate cancer immunity (25). The STING pathway is involved in the response to infection (26–28), inflammatory disorders and autoimmune diseases (29–32), and now is appreciated to play a role in the generation of antitumor immune responses (33).

As described previously, a sustained and effective antitumor immune response requires that antigen-presenting cells capture tumor antigen to prime and activate naïve T cells. Tumor-specific effector T cells must then traffic to and infiltrate into the tumor mass, subsequently recognize tumor antigen, and carry out cytolysis (the “cancer-immunity cycle”) (10, 11). However, in some tumor types, a paucity of neoantigen load, low immunogenicity, and/or minimal repertoire of neoantigens preclude sufficient T cell responses. These factors, as barriers to effective antitumor immune responses, and hence to the incorporation of immune-modifying therapies, becomes especially applicable in pediatric solid tumors, which are known to have low tumor mutational burden compared to adult tumors (34). This leads to a paucity of neoantigen formation on which to build competent immune responses, in particular for checkpoint blocking approaches. Additionally, many pediatric tumors express low levels of major histocompatibility complex (MHC)-I thereby further limiting their immunogenicity. Furthermore, children themselves are unique from an immunologic standpoint. Many solid tumors occur in young children, who have not yet had the typical number of immune-activating environmental exposures, and some of whom are not even yet fully vaccinated against typical pathogens such as hepatitis, measles, and varicella. This suggests that these tumors are occurring in an immunologically “immature” environment without the memory cell repertoire that would be expected in an adult cancer patient.

A picture of the tumor immune microenvironment is emerging for each of the major extracranial pediatric solid tumor types (NBL, Wilms’ tumor, hepatoblastoma, soft tissue sarcomas and bone sarcomas). However, a more detailed understanding of each tumor type and the complex interplay between the tumor cells, stroma and infiltrating immune cells will be necessary to realize the full potential for immune-modifying therapies in these tumors (35). Current evidence indicates that activated cytotoxic T cells and natural killer (NK) cells are present in some tumors in small numbers and that dendritic cells are also present, suggesting that all of the machinery necessary to mount a tumor-targeted cytolytic immune response, and for the generation of immunologic memory, are present within the microenvironment pediatric solid tumors (34). As more pediatric immunotherapy-focused pre-clinical experiments and clinical trials are completed, a more detailed picture of these factors will come into focus (36).

### 2.1 Neuroblastoma

NBL is the most common extracranial solid tumor in children. Despite primary tumor resection and multimodal therapy, children with metastatic or *MYCN*-amplified NBL experience a 45% risk of metastatic relapse and 30% risk of death; fewer than 10% of those with localized disease suffer relapse and death (37). Patients are stratified into high-risk (HR) and non-HR categories based on age at diagnosis, tumor stage, histology and genetic factors such as amplification of the *MYCN* gene. Immunophenotyping of NBL suggests cellular composition correlates with overall survival both in the entire cohort of patients (38–40) and even within the HR subset (41–43), and scores of cytolytic activation based on expression of perforin and granzyme independently correlate with overall survival (44). At the same time, immunohistochemical localization studies have shown that cytotoxic CD8^+^ T cells and NK cells are often excluded from the immediate vicinity of tumor cells, relegated to the tumor periphery or within fibrous stroma (45, 46). Furthermore, the relative frequency of suppressive cell types such as myeloid-derived suppressor cells (MDSC) and tumor-associated macrophages (TAM) increase while NK cell frequency decreases over time with NBL growth (47), and the phenotype of tumor-infiltrating CD8^+^ T cells is different from matched samples of peripheral blood CD8^+^ T cells in patients, with the tumor-infiltrating cells acquiring an effector memory phenotype, identified by the cell surface markers CD25, CCR7, and CD45RA (48). Taken together, these data suggest that the tumor cell and immune cell interactions contribute to the overall course of tumor development and outcome in NBL.

*MYCN* is a major genetic contributor to NBL clinical behavior, and half of patients with HR disease carry amplifications of *MYCN*. The TARGET (Therapeutically Applicable Research to Generate Effective Treatments) database contains transcriptional data from RNA sequencing of 149 NBL samples. TARGET database analysis showed that *MYCN*-non-amplified samples had higher infiltrating immune cell scores. CIBERSORT identified that infiltrating cells consist of activated NK cells and CD8^+^ T cells. Interestingly, *MYCN*-non-amplified samples had lower tumor mutational burden than the *MYCN*-amplified samples, and also had higher infiltrating immune scores, suggesting that immune infiltration of NBL is more dependent on its *MYCN* status than on mutational burden alone, as is traditionally thought for antigen generation. Furthermore, *MYCN*-amplified samples expressed lower HLA class I in both TARGET and validation cohorts (44). Transcriptional analysis of the SEQC cohort of 498 primary NBL tumor samples demonstrated lower T cell gene signature and lower cytotoxic immune cell gene signature in *MYCN*-amplified samples compared to *MYCN*-non-amplified. CIBERSORT analysis showed lower CD8^+^ T cell and higher regulatory T cell (Treg) cell infiltration in *MYCN*-amplified subset of tumors, with relatively constant monocyte and macrophage populations (49). *MYCN* directly modulates immune cell infiltration to the TME by altering cytokine production in NBL tumor cells. *MYCN* overexpression leads to transcriptional repression of the chemokine CCL2 and this decreased natural killer T (NKT) cell migration to human NBL xenografts in NOD/SCID mice. *MYCN* knockdown in high-expressing cell lines (SK-N-BE(2) and LAN-1) increased CCL2 mRNA transcript expression levels (50). Lower IL-15 and NKT cell immunoscores were found in *MYCN*-non-amplified samples in multiple databases of transcript expression in NBL, and IL-15 expression confirmed by RT-PCR in a separate sample of clinical cDNA NBL specimens (51). *MYCN*-amplified samples express high levels of the H3K9 histone-lysine methytransferases EHMT and EZH2, which directly contribute to low expression of CXCL9 and CXCL10 and a non-T cell-inflamed phenotype in primary tumor transcriptomic data as well as in human NBL cells, and can be reversed by targeted EHMT and EZH2 inhibitors (52). *MYCN* status will need to be considered when identifying which patients may derive maximum benefit from an immune preservation agent around the time of either radiation therapy or surgery.

STING activation of IFN production is closely linked to dendritic cell recruitment, maturation and antigen presentation of multiple tumor types. There is only sparse data on the frequency and role of dendritic cells within the primary tumor mass of NBL. In the transcriptional analysis of the SEQC database of primary NBL tumors, the dendritic cell marker CD141 was highly expressed in tumors with high CD3 transcript levels, and these two markers, together with the NK cell markers NCR1 and NKp46 co-localized within tumor samples when evaluated by immunohistochemistry (39). In another transcriptional study, both activated and conventional dendritic cells were amongst the cell types that correlated with prolonged patient survival (40). Conditioned media from two human NBL cell lines (NLF, GOTO) induced a tolerogenic dendritic cell phenotype (decreased CD1-α, IL-12, TNF-α, increased IL-6 and IL-10) and these dendritic cells were unable to activate cytokine production from invariant NKT cells *in vitro* (53). More will need to be ascertained regarding the contribution of dendritic cells to the growth and metastasis of NBL, as well as an understanding of how dendritic cell populations change in the context of systemic stress.

Attempts to modulate the tumor-associated immune components have shown efficacy in pre-clinical models of NBL. Combination immunotherapy with anti-CTLA4 checkpoint blockade, CpG, and anti-CD40 treatment led to regression of established syngeneic NBL tumors in the 9464D-C57BL/6 model, induced immunologic memory that protected against tumor re-challenge, and led to lower prevalence of Treg cells and higher CD8:Treg ratio in the TME (54). Combination therapy with anti-PD-L1 and anti-CTLA4 increased effector T cell and F4/80+ macrophage populations in tumor draining lymph nodes of a transgenic murine model, and combination therapy also protected against tumor re-challenge in a syngeneic model (47). PD-L1/PD-1 targeted checkpoint blockade increased CD8^+^ T cell and CD11c^+^/MHC-II^+^ dendritic cell infiltration in syngeneic Neuro-2a/AJ mouse model (55). Lenalidomide is able to reverse the suppression of IL-2-induced NK cell activation by NBL tumor cell and monocyte co-culture (56). Coupling of a dominant-negative TGF-β receptor to NK cell activating receptors led to improved tumor control and overall survival in NBL tumor-bearing NOD/SCIDγ mice after adoptive transfer of the engineered NK cells (57). CSF-1R blockade with BLZ945, a small molecule inhibitor, decreased macrophage infiltration to the TME and increased sensitivity to cyclophosphamide and topotecan in xenograft model in NOD/SCID mice (43). Adoptive transfer of human γ∂-T cells primed with zoledronic acid induced MHC-I expression and CXCL10 levels in the TME and reduced tumor growth, leading to increased overall survival in xenografts of SH-SY5Y human NBL cells in female athymic nude Balb/c mice (58). Intracellular delivery of cGAMP, the activating molecule for STING and IFN gene activation, increased release of damage associated molecular pattern (DAMP) molecules and pro-inflammatory cytokines CXCL10, IL-12, IFN-β and TNF-α, as well as decreased the Treg cell population and increased the CD8^+^ T cell fraction, leading to slowed tumor growth and improved OS in a syngeneic murine model of NBL. Furthermore, STING activation protected against tumor re-challenge in previously exposed animals indicating induction of a memory effect (59). These data suggest that nearly all of the available approaches to immunotherapy, including activation of the STING pathway leading to interferon production, are capable of affecting NBL tumor growth, and therefore have the potential to be useful agents when employed in combination with locoregional therapies to counteract the effects of the locoregional therapy on systemic immune activation in NBL.

### 2.2 Wilms tumor

Wilms’ tumor is the most common kidney cancer in children, and is generally characterized by excellent outcomes. In 15% of patients, metastatic disease, predominantly to the lungs, is present at the time of diagnosis. Major operation for the primary tumor is not delayed despite the presence of metastatic disease according to COG protocols, therefore most children with metastatic disease undergo a major stress event (nephrectomy) prior to any exposure to systemic therapy for metastatic disease control. In this context, immunotherapy applied at the time of the operation would be the initial therapy seen by metastatic tumor cells. Current knowledge of the immune component of the TME in Wilms’ tumor is limited. CD4^+^ and CD8^+^ T cells are both present in the TME and expressed markers of activation (60). The frequency of CD8^+^ T cells was lower in tumors >4 cm, those with invasive margins into the renal sinus, kidney capsule or ureter, and those that had nodal involvement or anaplasia, and lower CD8^+^ positivity correlated with a shorter disease-free interval after initial therapy (61). In a large cohort of 38 pathologic specimens, tissue microarray analysis demonstrated a paucity of CD3^+^ cells and they were excluded from the tumor cells into the tumor periphery and stroma, and a CD68^+^ macrophage population predominated (62). In a separate study, CD163^+^ macrophage density was different based on whether the tumor cells had blastemal, epithelial or stromal histology (63). Finally, tumor cells were capable of inhibiting NK cell cytotoxicity as well as decreasing their cytokine production and expression of activating receptors (64, 65). Though sparse, these data suggest that immune cell infiltrates vary by tumor cell histology and are actively regulated by the tumor cells, opening the opportunity that modulation during times of systemic stress could alter the dynamics of metastatic tumor growth and perhaps prime the metastatic cells for subsequent cytotoxic therapy. Interestingly, lung metastatic disease that does not rapidly respond to cytotoxic therapy is ultimately managed with whole lung irradiation, thereby presenting a second opportunity for immune-modulation after initial therapy.

### 2.3 Hepatoblastoma

Hepatoblastoma presents with highly divergent histologic patterns and is frequently metastatic. The majority of work has centered around identifying and characterizing molecular drivers and subtypes, such as *CTNNB1*-mutated hepatoblastoma, or that occurring in patients with germline antigen-presenting cell mutations. Hepatoblastoma occurring in patients with germline antigen-presenting cell mutations have lower grade tumors with higher frequency of fetal histology (a positive prognostic factor), absence of CTNNB1 mutations and better clinical outcomes. Tumor samples taken after cisplatin therapy in these tumors demonstrated robust tertiary lymphoid structure generation within tumors, and higher CD3^+^ and CD8^+^ T cell populations (66). Similar to NBL and the other pediatric solid tumors, hepatoblastoma generates a low neoantigen load. A low tumor mutational burden was identified in 31 tissue samples of refractory and metastatic hepatoblastoma, with an average of 3.5 mut/Mb. *CTNNB1* was the most frequently mutated gene, present in 61% of samples, and no mutations in *TP53* or DNA repair pathways were identified (67). Despite this, CD8^+^ T cells are present within the TME, though at a much lower rate than either NBL or rhabdomyosarcoma (68). Hepatoblastoma tumor cells produce cytokines (66, 69) as well as innate immune receptors that can inhibit tumor cell migration and cytokine production when activated (70). Furthermore, innate-like γ∂-T cells can target and kill HBL tumor cells either with the aid of an EpCAM-targeted antibody (71) or by combination therapy with bispecific EpCAM/CD3 antibody and histone deacetylase inhibition (72). Much more data will be needed to ascertain the composition of immune cell infiltrates within the hepatoblastoma TME, especially in the context of metastatic disease, who can achieve transplant candidacy and subsequently are immunosuppressed to prevent organ rejection, raising the potential for immune escape mechanisms to play a major role in relapse.

### 2.4 Soft tissue sarcomas

Childhood soft tissue sarcomas are largely categorized based on their chemo- and radiosensitivity, into rhabdomyosarcoma and non-rhabomyosarcoma soft tissue sarcomas (NRSTS). Surgical resection plays a large role in the majority of NRSTS as these are generally chemoresistant tumors and resection gives the only chance at long term cure. Novel approaches to therapy will be critically important for these tumor types, especially as it relates to the perioperative period, as nearly all children who are managed with curative intent will undergo an operation during their therapeutic course. In a primary murine soft tissue sarcoma model, the TME was dominated by M2-polarized macrophages with low activated CD8^+^ T cell population. This was consistent 4 out of 5 “sarcoma immune class” categories of human primary tumor samples, comprising a majority of human tumors (73). In another study of ten tumor samples of infantile fibrosarcoma demonstrated markedly higher CD4^+^ and CD8^+^ T cell infiltration (40-50x) compared to age- and sex-matched rhabdomyosarcoma tumor samples, and fusion negative tumors contained a much higher fraction of activated CD8^+^ T cells (74), and rhabdomyosarcoma samples generally exhibit low CD3^+^ lymphocyte frequency with predominant macrophages, which correlate with improved event-free and overall survival (75), though tertiary lymphoid structures do form in rhabdomyosarcoma tumors (76). Checkpoint molecules are expressed in most rhabdomyosarcoma samples and half contain tumor-infiltrating lymphocytes with the cognate receptor (77). In an interesting study that tested timing of checkpoint therapy administration, targeting PD-1 is only effective when given very early after tumor initiation, unless CXCR2 signaling is blocked; with CXCR2 blockade, there is lower myeloid-derived suppressor cell infiltration into the TME, which restores the efficacy of subsequently initiated anti-PD-1 therapy against tumor growth (78). This suggests that timing of immunotherapy administration is critical to effectiveness, and likely represents a paradigm by which cellular infiltrates change over time with tumor development or in relation to stressful stimuli and thereby affect susceptibility to various therapeutic agents.

### 2.5 Bone sarcomas (osteosarcoma and Ewing sarcoma)

Osteogenic sarcomas are typically the least sensitive to systemic therapies, and local control with surgical resection is the single most important factor to achieving a long-term remission. Similar to soft tissue sarcomas, high rates of checkpoint molecule expression are found in osteosarcoma (77), though checkpoint inhibition has not shown efficacy thus far (79). Both primary tumor and metastatic samples demonstrate activated lymphocytic infiltration as well as mixed infiltrates of suppressive cell types such as TAMs, MDSCs, and Tregs (80–82). Tumor cells can induce suppressive-type TAMs with suppressive cytokine production (IL-10, TGF-β and CCL22) which can be reversed with TGF-β blockade (83) and hepatocyte growth factor blockade improves infiltration of a GD2-targeted CAR-T cell construct (84), suggesting that the tumor cells regulate immune cell infiltration and phenotypic differentiation within the TME. Furthermore, high USP6 expression correlates with improved survival in Ewing sarcoma. USP6 promotes IFNAR expression at the cell surface and CXCL10 and CCL5 chemokine production, leading to increased infiltration and activation of NK cells, dendritic cells and TAMs *in vivo* in the TME (85). Similar to other tumor types, though data is limited, infiltrating immune cells show some ability to react to tumor cells and are also modulated by the same, suggesting that disrupting this cycle or further boosting the infiltrating cellular population could take advantage of targeted cytolysis and improve tumor control in these otherwise hard to treat tumors.

### 2.6 Metastases

Pediatric solid tumors demonstrate typical locations for metastatic disease that is dependent on tumor type: lung metastases predominate for bone and soft tissue sarcomas, Wilms tumor and hepatoblastoma, while metastatic neuroblastoma overwhelmingly presents with bone marrow infiltration. There is a paucity of direct data regarding the immune phenotypes of metastatic lesions in contrast to primary lesions for these pediatric solid tumors. Osteosarcoma pulmonary metastases demonstrate an “immune-excluded” phenotype, with both potentially productive though suppressed TILs and immunosuppressive myeloid cells present along the periphery of lesions, and transcript levels of the respective anti-tumor and pro-tumorigenic immune cell subtypes in these metastatic lesions were associated with differences in progression-free survival (82). Bone marrow is the primary metastatic site for NBL, and a thorough review of the literature did not identify clear differences in either lymphoid or myeloid cell populations in NBL-infiltrated bone marrow, however they highlight that limited data are available directly addressing the interactions in bone marrow amongst these cell types and with tumor cells (86). Interestingly, immunotherapies have been demonstrated in a pre-clinical NBL model to eradicate metastatic bone marrow disease (87), suggesting that the immune microenvironment at the metastatic location could be important to tumor cell persistence and progression.

## 3 Systemic responses to surgery and radiation therapy

In addition to local immunosuppression within the tumor, patients with cancer have systemic immune compromise evidenced by increased incidence of infection and attenuated response to immunizations (88, 89). Tumor development weakens the systemic immune landscape, and local recurrence of tumor burden after resection is sufficient to reduce the capacity for optimal T cell function (90). Thus, anticancer approaches targeting immune-oncologic pathways must be considered in the context of both local and systemic action.

Prior to the introduction of immune-modulating agents into the repertoire of cancer therapeutics, cancer treatment options consisted of systemic chemotherapeutic agents, focal radiation therapy, or surgical resection of macroscopic tumor burden. The latter two modalities are primarily used as tools to treat specific areas of focus, rather than with an intent to induce systemic responses. However, historical observations have demonstrated the widespread biological effects of locoregional therapies.

### 3.1 Immune responses to surgery

**Figure 1:**
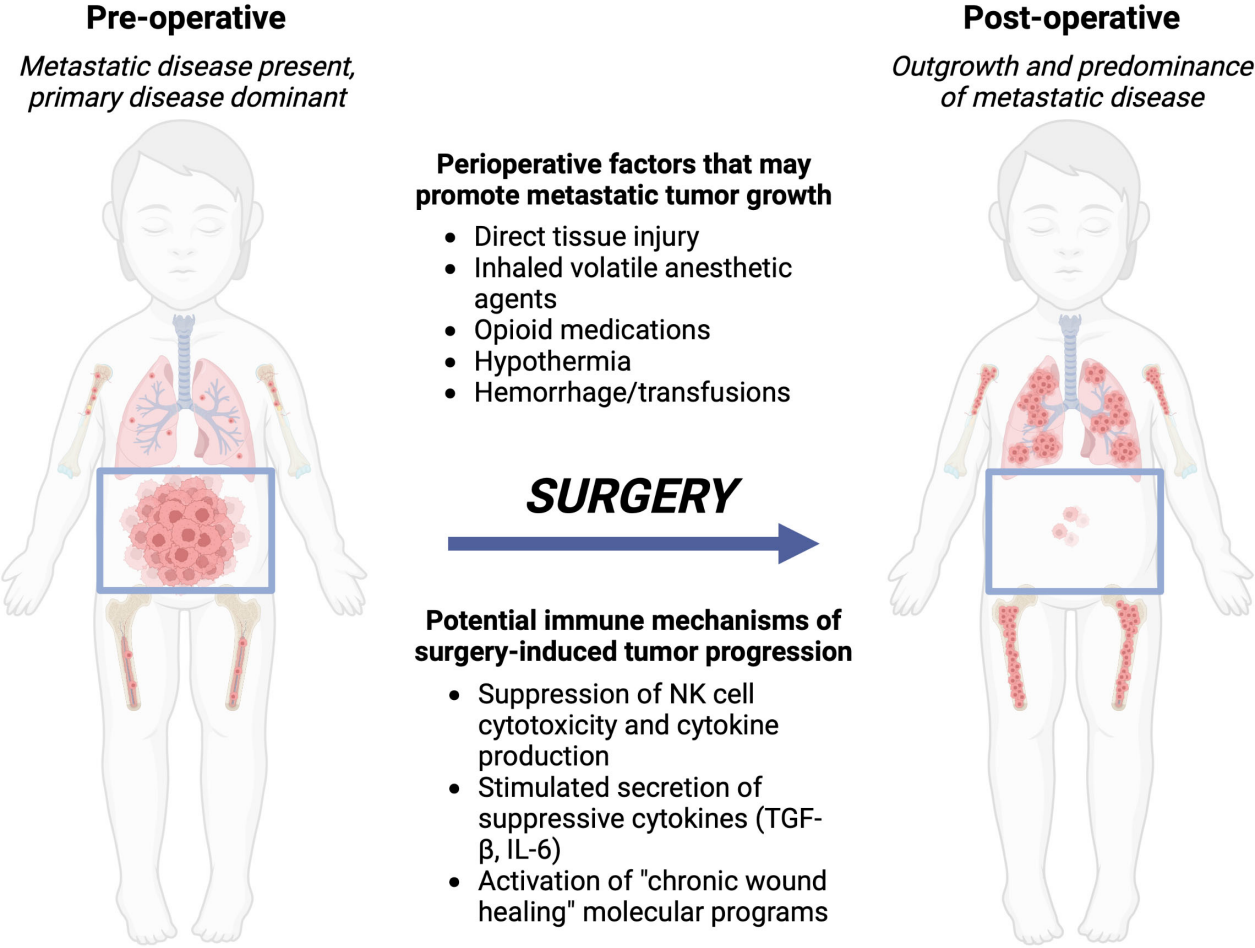
Local control with surgery or radiation of the primary tumor can lead to metastatic outgrowth of distant disease. *Left*, the preoperative patient with a bulky primary abdominal tumor and distributed metastatic disease. *Right*, after surgery, the primary tumor is removed, however, metastatic disease progresses due to immune-mediated changes from the perioperative factors a patient is exposed to during and around the time of surgery. Created with BioRender.com.

During and after an operation, multiple neurohormonal systems are activated by a variety of individual stimuli: the local tissue trauma of the operation itself; hypothermia, blood loss, and pain that often accompany an operation (5); and systemic modulation of the immune system distant from the actual site of tumor removal (7, 91, 92). We and others hypothesize that these stimuli conspire to alter the behavior of residual tumor cells that are not removed at the time of operation. Residual disease can persist after an operation because of incomplete primary tumor resection, shedding of tumor cells into the circulation while handling the primary tumor during removal, or due to distant micrometastatic foci that are already present at the time of the operation. These tumor cells are then exposed to direct modulation by the perioperative neurohormonal signals and also to indirect modulation by the immune system, which may lead to recurrence if surgery is utilized as the definitive therapy, or may lead to resistance to subsequent, conventional adjuvant chemo- or immunotherapy (**Figure 1**). In this section, we will review pre-clinical experimental data and clinical trial data that demonstrate a net “pro-tumorigenic” effect of an operation.

Two robust pre-clinical models have been established that inform our understanding of the direct surgical and perioperative effects on the immune system and tumor behavior. In one model surgical stress was induced in wild-type Fischer 344 rats by performing a midline laparotomy, followed by exteriorization of the intestine, gentle rubbing of the intestinal serosa with damp gauze and replacement of the intestines to the peritoneal cavity, for a total operative time of 1 hour (93–99). Surgical stress in this model increased susceptibility to lung tumor formation when the animals were challenged 5 hours post-operatively with intravenous injection of MADB106 rat mammary carcinoma cells, which are syngeneic and non-immunogenic (93–95, 98).The second model induces surgical stress in wild-type Balb/c and wild-type C57BL/6 mice by performing a 3 cm midline laparotomy followed by either left hepatectomy or left nephrectomy. Syngeneic murine CT26LacZ colorectal cancer cells or 4T1 mammary carcinoma cells (used in the Balb/c mice), and B16F10LacZ melanoma cells or MC38 colorectal carcinoma cells (used in the C57BL/6 mice) were given intravenously prior to the operation and lung metastatic formation was assessed (100–104). Similar to the previously established rat model, surgical stress in the mouse model increased lung metastasis formation with each of the tumor cell types (100). Both models (rat and mouse) have been utilized to examine both the neurohormonal mediators of the surgical stress response as well as the specific immunologic consequences of the stress responses.

The major stress-mediating systems include catecholamines, prostaglandins and glucocorticoids, which are differentially linked to various stimuli and lead to differential downstream system activation. Increased susceptibility to lung metastasis formation after surgical stress in the rat model was partially reversed by pharmacologic blockade of cyclooxygenase (indomethacin or etodolac) or beta-adrenergic receptors (nadolol or propranolol) in a dose-dependent manner (93–95, 98). Prostaglandin E2 administered to wild-type rats who were not exposed to surgical stress increased lung tumor formation, mimicking the effect of surgical stress. Prostaglandin-induced increased tumor formation was similarly reversed by indomethacin (94).Surgical stress increased plasma corticosterone and IL-6 levels and this effect was reversed by the cyclooxygenase inhibitor indomethacin (93). Taken together, these data highlight that both the catecholamine and prostaglandin hormonal systems feed into the observed effects of surgical stress on susceptibility to metastasis formation, however more data are needed to discern the specific impact of these systems on cellular subsets of the immune system within the context of surgical stress-induced activation, in order to design effective therapeutic strategies to modulate this response to operations.

Immune responses to surgical stress are inherently mediated by the various cell types and subtypes that comprise both the innate and adaptive systems. Data from the two major pre-clinical models that have been discussed point toward an integral role for NK cells in this process. Transfer of NK cells from a surgically stressed animal to a non-stressed, NK cell-deficient animal led to increased lung tumor formation compared to transfer of non-stressed NK cells (103). NK cell depletion diminished the surgery-induced increase in lung metastasis formation in the Balb/c mice when challenged with either the CT26 colon cancer or B16F10 melanoma cells (100). Surgical stress impaired NK cell cytotoxicity in the subset of NK cells present in the lung without causing the same effect in the circulating NK cell population. Cyclooxygenase (COX) inhibition reversed this effect (93–95), in an isoform specific manner with COX-2, but not COX-1 inhibition, mediating the effect (98). However, little is known about the responses of T cells, B cells or specific myeloid populations in the surgical stress response context. Cellular immune responses can be defined either by number of cells of each type present after a given stimulus or within a specific organ system or in the peripheral blood, as well as through their effectors functions such as cytolytic capability and cytokine production. Surgical stress increased neutrophil counts in both lungs and blood by 2.5-fold, and decreased peripheral circulating T cell counts by 65% (99). Surgical stress in the murine model increased the circulating levels of IL-5, IL-6 and TGF-β with no effect on chemokines. The MDSC population in the spleen increased after surgery, and the levels of NK cell activating ligands NKG2D and KLRG1, and the T cell adhesion molecule CD62L all decreased in the spleen after surgical stress, suggesting a shift toward an overall suppressed systemic immune state (103).

Another approach that reveals the role of the cellular immune responses to surgical stress is the effect of immune-activating pharmacologic agents during the perioperative period. Administration of the TLR3 agonist poly(I:C) at low dose for 5 days before the operation blocked the surgical stress-induced increase in experimental lung metastasis formation, and also blocked the suppression of NK cell cytotoxicity after surgery. Poly(I:C) also protected lung-specific NK cells, but not a general peripheral blood leukocyte population, from *in vitro* suppression of cytolytic capability from corticosterone or prostaglandin E_2_ (PGE2) exposure (96, 97). Similar to the rat model of surgical stress, peri-operative administration of TLR3 agonist poly(I:C) reduced susceptibility to surgery-induced lung metastasis formation in both the colorectal and melanoma murine models, and also reduced the effect of surgery on NK cell dysfunction (104) and also protected animals from a sepsis-induced increase in lung tumor formation (102). Pre-operative administration of IL-12 at low dose increased resistance to experimental lung metastases after surgical stress and also increased the total load of lung-specific NK cells thereby increasing their total cytotoxicity. NK cell depletion did not change the anti-tumor effect of IL-12 administration, suggesting that additional mechanisms may mediate the IL-12-associated resistance to metastasis formation (99).

Very few studies have specifically examined the immune responses following an operation for cancer. Four clinical trials have been completed by the same two research groups that established the previously discussed pre-clinical models, which reveal consistent findings with the pre-clinical work, and suggest that pre-clinical models provide value to inform the design of potentially translatable therapeutic interventions for the perioperative period. The first two trials we review here involved peripheral blood sampling to identify cytokine and cellular responses to a cancer operation. A cohort of 59 patients undergoing either major or minor-intermediate operations were assessed for immune responses after surgery by peripheral blood sampling and compared to 22 healthy controls who did not undergo an operation. Plasma IFN-γ and IL-6 levels increased after the operation, while induced cytokine production (IFN-γ, IL-6, IL-10, IL-12) was inhibited suggesting circulating immune cell dysfunction in the presence of increased circulating pro-inflammatory mediators after surgery. Additionally, the numbers of circulating NK cells/mL of blood were reduced after surgery as was their cytolytic activity (105). Multiple changes in circulating immune cell populations were identified in the surgery cohort. Granulocyte counts were increased, while populations of cytolytic, helper, Tregs, NKT and NK cells were all decreased after both major and minor-intermediate operations. Furthermore, surgery decreased MHC-II expression on both lymphocytes and monocytes, indicating potential decreased antigen sensitivity after an operation even for the cells that are present (106). A separate cohort of 42 patients with colon cancer had blood collections performed on days 1, 3, 5, 28 and 56 after their operation, and compared with 27 healthy donor blood samples. The cancer patients showed impaired cytokine production by NK cells at baseline compared to the healthy controls, and this further reduced up to 83% post-operatively. A third of cancer patients had persistent impairment of cytokine production at 56 days after surgery. NK cell number was not altered at any point post-operatively in this study. A brief increase in CD14^+^ monocytes was observed at day 1 after surgery, however this had returned to normal by 3 days post-operatively (107).

In the next two trials, pharmacologic interventions that modulate the stress-mediating neurohormonal systems demonstrate consistency with the pre-clinical experimental data. Combined beta-blockade (propranolol) and COX blockade (etodolac) were given in a randomized controlled trial to patients with early stage breast cancer for 5 d pre-operatively and continued for 6 days post-operatively. Dual blockade reduced pro-metastatic and pro-inflammatory transcription factor expression in tumor samples, and reduced post-operative increases in serum levels of IL-6 and CRP. Induced cytokine production from blood samples was restored after the dual blockade therapy and CD14^+^/CD16^-^ monocyte populations were less predominant in peripheral blood compared to untreated controls. Furthermore, dual blockade increased CD11a^+^ expression on NK cells (108). In a separate trial, combined propranolol and etodolac therapy in patients with colon cancer were administered for 5 days pre-operatively and continued for 15 days post-operatively and tested in a double-blind, randomized format against placebo control. Tumors were subjected to bulk mRNA expression analysis at the time of resection, and patients were followed for clinical recurrence of disease. Treated patients showed less CD14^+^ monocyte and CD19^+^ B cell infiltrates and increased CD56^+^ NK cells, while dendritic cells, CD4^+^ and CD8^+^ T cells were no different. For patients who adhered to the treatment protocol, recurrence rate trended lower (0%) at three years compared to the placebo group (29.4%) (109).

### 3.2 Immune responses to radiation

Growing evidence demonstrates that radiation therapy (RT) acts as a stimulus for widespread biological effects beyond the treatment field receiving direct radiation. Early data in 1979 demonstrated that tumor radiosensitivity relies on competent host immune system (110). More recent data from melanoma mouse models have demonstrated that ablative RT reduces tumor burden in a CD8^+^ T-cell-dependent manner and increases priming of tumor antigen-specific T cells at tumor-draining lymph nodes (111). Furthermore, inherently immunogenic tumor types responded to RT more robustly than non-immunogenic tumors. Largely, stimulation of antitumor immunity by RT occurs *via* direct effects on tumor cell signaling, induction of immunogenic cell death, and modulation of immune cell signaling. RT-induced DNA damage culminating in type I IFN responses is one mechanism for stimulating antitumor immunity. Ablative RT increased the production of IFN-β within tumors both by cancer cells and inflammatory cells of the TME (21, 112). Furthermore, type I IFN was required for RT-induced dendritic cell activation and tumor control (21). Of the several pathways upstream of IFN-I production, STING was the essential regulator of RT-induced IFN-I-dependent generation of adaptive immune responses and antitumor effects (113). Moreover, double-stranded DNA within exosomes of irradiated tumors acted as an adjuvant by stimulating surface expression of costimulatory molecules and expression of *IFNB1, MX1* and *IFNAR1* genes by CD11c^+^ dendritic cells in a STING-dependent fashion (114). cGAS, the enzyme responsible for generation of endogenous STING ligands, was indeed shown to mediate dendritic cell sensing of irradiated tumor cells, suggesting that stereotactic radiation techniques may depend on host STING and the downstream type I IFN driven response (113).

Until the discovery of the immune system’s contribution to tumor cell killing in response to RT, RT was largely viewed as immunosuppressive. This view was in part due to the radiosensitivity of some immune cells. Indeed, venous blood samples were collected from a cohort of lung cancer patients receiving conventional chemoradiotherapy and showed that absolute lymphocyte count within peripheral blood decreases over the course of treatment, although the relative abundance of lymphoid versus myeloid cells compared to baseline does not change (115). Treatment-related lymphopenia was examined in a computational glioma model concluding that potentially lymphotoxic radiation doses are delivered to much of the circulating blood pool over the course of standard treatment (116). Immunosuppressive adaptations can arise within irradiated tumors. Free radical species that can be generated by RT upregulate factors within tumor cells that promote survival in hypoxic environments. Hypoxia a key hallmark of cancers, and can mediate immunosuppressive signals (117). RT was found to induce STAT3 activation thereby increasing MDSC infiltration into the TME of pancreatic tumors to mediate post-RT immunosuppression (118). Single dose RT at 20 Gy increased CCR2-dependent MDSC infiltration following radiation as a mechanism of radioresistance in the tumors (119). In a murine model, TAMs from irradiated tumors increased expression of immunosuppressive genes and increased the tumorigenicity of co-cultured tumor cells (120). Tumors with an abundance of macrophages are more radioresistant, underscoring the importance of the biology of the tumor and the characteristics of the TME as factors in determining response to RT (121, 122). The T cell compartment can also contribute to resistance mechanisms; PD-L1 was upregulated in irradiated tumors in response to IFNγ from effector T cells (123). Furthermore, Tregs are inherently radioresistant (124, 125). Treg infiltration was observed in relapsed tumors following RT and immune checkpoint inhibitor treatment, and depletion of these immunosuppressive cells restored antitumor immunity (126). These examples of RT-induced immunosuppression suggest that targeting these pathways in a combinatorial treatment strategy will potentiate the antitumor effects of RT and allow for sustained tumor antigen-specific immune responses.

RT dose impacts the immune response outcomes. Many studies indicate that modulation of the immune system by low-dose, conventionally fractionated radiation is largely associated with pro-tumorigenic effects (127). High-dose ablative RT that directly induces cell death and subsequent release of DAMPs from dying cells is more frequently associated with immune activating signals that culminate in the generation of adaptive immune responses (128). Indeed, dose-dependent activation of the cGAS/STING/IFN-I pathway in response to radiation (112). The abscopal effect describes untreated tumor sites that are responsive to the radiation of a distant tumor. When RT is used alone, the abscopal effect is observed only rarely (9). While RT enhances tumor immunogenicity and immunogenic cell death, regulatory mechanisms are simultaneously induced that impede on its ability to effectively act as an *in-situ* vaccination and induce antitumor immunity as a single therapeutic modality.

Pattern recognition receptor (PRR) agonists have been shown to potentiate the immune stimulating effects of RT. TLR9 or TLR7 agonists in conjunction with RT synergistically amplified the antitumor response in preclinical models (129, 130). In a phase I/II clinical trial, TLR9 agonist CpG increased abscopal response rates (131). STING agonist treatment in a colon carcinoma model after single dose RT ablated tumors in approximately 70% of mice compared to 0% in either treatment alone, and increased the number of tumor antigen-specific T cells in the draining lymph nodes (113). In a pancreatic cancer model, STING agonist and RT similarly synergized to decrease tumor burden and improve survival (132). Combination therapies with RT and already-in-clinic ICI biologics are also being tested. A multi-dose regimen of RT and ICI induced antitumor immunity at both irradiated and unirradiated sites whereas a single RT dose with ICI did not in a preclinical model (133). Low-dose targeted radionuclide therapy with ^90^Y-NM600, an alkylphosphocholine analog that semi-selectively accumulate in most tumors, synergized with checkpoint inhibitor therapy to induce a complete response in the majority of mice bearing immunologically inert tumors, including in a NBL model, in a STING-dependent manner (134). This study exemplifies the importance of immune-activating signals in the TME for the success of antitumor effects of RT and T cell-targeting immunotherapies. There are multiple ongoing Phase I, II and III studies investigating the safety and efficacy of combined RT and checkpoint inhibitor approaches, the emerging results of which have been reviewed elsewhere (135). Since the introduction of immunotherapies as a pillar of cancer therapy, there has been an increased incidence of abscopal effects in patients dually treated with RT, demonstrating the success of immunotherapy and locoregional therapy synergy (136, 137).

## 4 Immune activating agents as anticancer therapeutics

Multiple classes of anticancer therapies depend on intact type I IFN signaling for their efficacy, including monoclonal antibodies, cytotoxic chemotherapy and radiation therapy. Therapeutic strategies to activate type I IFN signaling include immunocytokines, adoptive transfer of recombinant IFN-producing cells, vectors of recombinant DNA to encode production of type I IFN, and PRR agonists (23). The PRR cGAS-STING was identified as a necessary upstream mediator in the type I IFN-dependent generation of endogenous antitumor immunity in immunogenic tumor types, identifying the downstream STING pathway as a critical bridge to activate cancer immunity. Native cGAS-STING recognizes cytoplasmic double-stranded DNA, which indicates viral infection, or mitochondrial or nuclear damage that leads to DNA leak into the cytoplasm of the cell. The downstream inflammatory signaling pathway activated by this system includes TANK-binding kinase 1 (TBK1)-dependent interferon regulatory factor 3 (IRF3) activation to induce production of type I IFN, as well as activation of nuclear factor κB (NF-κB). Of the three major cytoplasmic DNA sensor PRR systems (TLR9, AIM2, cGAS), cGAS-STING is the major stimulator of type I IFN production. Some of the first exogenous STING ligands to be tested in clinical trials as anticancer agents were 5,6-Dimethyl-9-oxo-9H-xanthene-4-acetic acid (DMXAA) and mixed-[2,3]-linkage (*R, R)* cyclic-diAMP (ADU-S100). DMXAA failed in a clinical trial for non-small cell lung cancer and was later found to selectively target murine STING only, and was incapable of activating human STING, explaining the divergent pre-clinical and clinical trial results. ADU-S100 was strategically developed in 2015 as a potent STING agonist capable of binding all the five common human STING alleles as well as mouse STING (138). Local ADU-S100 administration has shown preclinical efficacy at inducing systemic antitumor immunity, reducing tumor size and improving survival as a single agent in mouse models of immunogenic tumors (138–141) and non-immunogenic tumors (142, 143) and is currently in clinical trials as a single agent (NCT03172936) or in combination with anti-PD-1 (NCT03172936 and NCT03010176). Results from a phase I trial assessed a range of intratumoral ADU-S100 injection doses (NCT02675439) and showed that, as a single agent, this STING agonist therapy was well-tolerated, displayed evidence of systemic immune activation in the peripheral blood, and reduced or maintained size of the injected lesion in 94% of patients; however, significant changes within the TME immune infiltrate were not observed at two weeks post-treatment and overall tumor burden response rates were limited (144).

Newer generation STING agonists have utilized multiple approaches to overcome challenges related to drug delivery to the TME, particularly the highly polar nature of native, cyclic di-nucleotide STING agonists. Various groups have implemented strategies to improve delivery and enhance uptake to tumor tissue including through nanoparticles and lipids (145–150). These have been extensively reviewed elsewhere (151, 152). A promising oral agent with pH-sensitive activation, MSA-2, was recently reported and may open new opportunity for practical and effective STING agonism in the clinic. In summary, the efficacy of STING agonist is mediated by IFN signaling and CD8^+^ T cell priming and recruitment (153). Effector T cell recruitment in response to STING activation is dependent on the CXCR3 chemokine axis, the ligands of which, CXCL9 and CXCL10, are upregulated in the TME (113, 138, 142, 143). STING agonists have demonstrated promising pre-clinical activity in models of both immunogenic and non-immunogenic tumor types and may represent a novel approach to counteract the surgery-induced suppression of antitumor immunity.

Non-STING PRR agonists have been extensively studied as potential anti-cancer therapies. Much of the focus has been on the TLR system, however, the only FDA approved TLR agonists for oncologic indications are Bacillus Calmette-Guerin (BCG), monophosphoryl lipid A (MPL), and imiquimod (154). These three agents have been approved for over ten years now and no other TLR agonists have been able to make the transition to approval for use over the same time period, despite an extensive number of clinical trials (155). In particular, the nucleic acid-sensing TLRs, TLR3, TLR7/8, and TLR9, have shown particular promise in clinical trials (156, 157) and were recently reviewed in depth elsewhere (158). The major challenge of the clinical data accumulated to date is that this approach has been tested predominantly in patients with treatment-refractory or unresectable primary disease. Tumors that already have demonstrated a poor response to conventional therapies may not fully represent the potential for these agents to serve in a useful capacity in another therapeutic context, such as serving as a complement to surgery to counteract the negative immune impacts of surgery on residual tumor burden. PRR agonism in pediatric solid tumors have been explored in a limited fashion and exclusively in pre-clinical experimental settings, with promising results. TLR3 agonism with poly(I:C) led to tumor regression in a mouse xenograft model for *MYCN-*non-amplified tumors but not *MYCN*-amplified tumor grafts (159), however when poly(I:C) was added to isotretinoin this did slow tumor growth in a *MYCN*-amplified xenograft model, and showed phosphorylation of IRF3 and induction of TLR3 and MAVS expression but not MDA5 or RIG-I *in vitro*, suggesting a TLR3-depedent mechanism (160). Poly(I:C) additionally increased MHC-I expression on multiple NBL cell lines and increased activation markers on CD4^+^ and CD8^+^ T cells in a co-culture model (161). TLR9 agonism with CpG oligonucleotide inhibited NBL cellular proliferation and prolonged survival in NBL tumor xenograft-bearing mice (162). CpG also increased sensitivity to radiation toxicity *in vitro* and combination therapy with RT protected against tumor re-challenge, indicating a memory effect (129). TLR4 activation with lipopolysaccharide decreased invasiveness of hepatoblastoma HepG2 cells *in vitro (* 70), and reduced *in vivo* tumor growth and metastatic progression in a syngeneic osteosarcoma model *via* a CD8^+^ T cell dependent mechanisms (163). These data suggest an overall promising potential for TLR agonists in pediatric solid tumors, demonstrating anti-tumor activity in a variety of pre-clinical experimental models. These agents have not yet been tried in a clinical trial in pediatric solid tumors.

## 5 Counteracting the suppression of anti-tumor immunity associated with surgery and radiation

**Figure 2:**
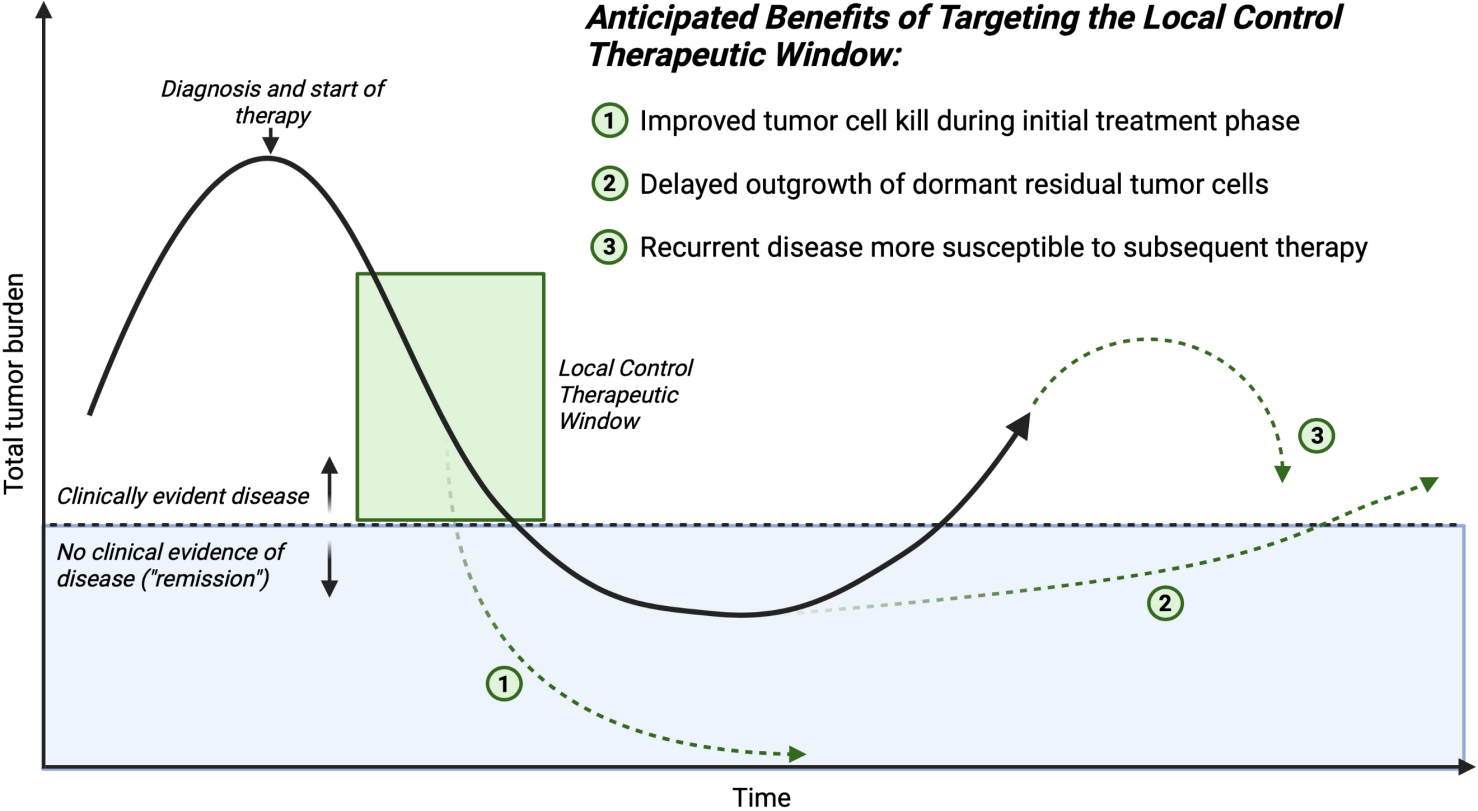
Time course of an individual patient’s disease process. The mechanisms of improving outcomes for children who require major surgery for cancer, and receive complementary therapy in conjunction with traditional local control (surgery or radiation) to reverse the negative systemic impacts sustained during the critical local control therapeutic window. Created with BioRender.com.

The potential translational benefit of STING agonists or similar agents such as TLR agonists as a complement to surgery stem from their hypothesized mechanism of action: increasing IFN-I production by cancer cells or accessory stromal cells within the tumor to increase activating cytokine production and to stimulate effective antigen presentation leading to preserved or augmented anti-tumor T cell responses, counteracting the systemic inflammatory signals from surgery or radiation that support recruitment and expansion of suppressive MDSC, TAM, and Treg to the tumor. We hypothesize several mechanisms by which clinical benefit to children with solid tumors would manifest from using this approach (**Figure 2**). Targeted immune activation could extend the net total debulking effect of surgery. If one considers total tumor burden as a continuous variable that is decreased by the collective contribution of all applied therapies during the induction and consolidation phases of management, increasing the presence and activity of cytotoxic effector T and NK cells within the distant tumor foci would contribute to the total decrease in tumor burden. This may increase the probability of complete elimination of all residual tumor cells including stem-like tumor cells (total tumor burden of 0, or true “cure”). Alternatively, lowering the residual disease burden at the end of therapy may delay or prevent emergence of resistant clones and eventual clinical relapse.

Shifting the immune cellular milieu of the TME may induce a more robust T cell memory response. This would be hypothesized to aid in the prevention of future relapse, even if microscopic residual disease persists over the long-term, because an antitumor immune response could be activated if a dormant stem-like tumor cell began a growth cycle. We hypothesize this potential effect would be most important in the cases in which surgery is the last antitumor therapy that a patient receives before entering a surveillance protocol.

Lastly, residual disease that persists after surgery may be sensitized to subsequent conventional adjuvant therapy. The ultimate tumor cell killing effect of cytotoxic chemotherapeutics is in some cases immune cell mediated, therefore shifting the immune balance in the TME away from a surgery-induced suppressive state would lead to increased efficacy of post-operatively administered chemotherapeutics. Taken together, these hypothesized beneficial effects of complementary immune activation at the time of surgery are multifaceted and could positively benefit patients through a number of different mechanisms. This potential broad impact suggests that further investigation of these hypotheses is urgently needed.

Prior to testing our overall proposed strategy in a clinical trial, several knowledge gaps need to be addressed with pre-clinical investigations. First, longitudinal tumor-specific histopathological and systemic immune profiling data are needed. Most existing data on the immune cellular composition of the TME in pediatric solid tumors comes from analysis of tumor samples taken at the time of resection. By looking at this timepoint alone, we potentially miss important information about the state of the tumor immune microenvironment at the time of diagnosis, changes that may occur in response to neoadjuvant therapies, and how the tumor looks at the time of recurrence. It is challenging to perform these longitudinal studies in patient populations, and therefore important knowledge could be gained through the development of immune competent animal models that reflect the full clinical course of the patient’s experience. This would necessitate survival surgical models in tumor-bearing animals that are also amenable to pre-operative and post-operative exposures with the same cytotoxic agents utilized clinically for each respective tumor type. Second, much more information is needed about the systemic immune “macroenvironment” for pediatric solid tumors. Much of the antitumor immune response is engineered outside of the TME proper, and so understanding these extra-TME locations (e.g. tumor-draining lymph nodes, spleen) that enable antitumor immune responses will be critical to implementing effective immune targeting strategies (90, 164). The systemic immune “background” is at the time of surgery for pediatric solid tumors is largely unknown. Attempts to inform this aspect of oncologic surgical care have been made using clinically available data through peripheral circulating cell counts collected prior to an operation. The most often utilized value is the neutrophil-to-lymphocyte ratio, which attempts to capture the relative balance of innate and adaptive immune status at the time of the blood sample collection (165, 166). Though widely studied in adult solid tumors, there is a scarcity of similar data for pediatric solid tumors. More sophisticated analyses of the systemic immune macroenvironment over time will in the near-term only be possible through pre-clinical work. Third, there is a paucity of data to inform the intrinsic antitumor capabilities of targeted immune activating agents against pediatric solid tumors. Positive impact against pediatric solid tumors generally, outside of the perioperative window, need not be a pre-requisite to consider use of these class of agents within the perioperative window, for reasons outlined in this Review. Fourth, targeted immune activation would ideally need to be restricted to the TME of residual disease, so as not to precipitate systemic immune response syndrome or impede the general recovery from the surgical procedure. Drug development efforts continue to progress for STING agonists, as indicated by a recent report of a pH-sensitive oral STING agonist agent that is inactive systemically and restricts its STING activation to acidic environments, which would target the TME of solid tumors (167). How applicable this type of approach would be for residual disease, much of which would likely be microscopic, remains to be determined. An alternative approach to achieve TME-specific targeting could be the existence of a wider therapeutic window to achieve the goal of counteracting the immune effects of surgery, which may require much lower doses of immune activating agents, compared to the doses that would be required to achieve a response outside of the perioperative window, which is the typical approach to nearly all clinical trials. Finally, identifying immune activating agents with limited adverse effects toward surgical recovery will be critical to capitalizing upon the potential benefit of this proposed approach in pediatric solid tumors. Systemic inflammatory responses to TLR or STING agonists pose a potential direct threat to the patient, as well as confounding the clinical assessment of either readiness for surgery, or recovery from surgery, such as determining whether or not an infection is present. Managing these potential adverse effects while not delaying or precluding effective surgical care has been demonstrated previously (168, 169) and has also been demonstrated in the use of immunotherapy more generally, such as with checkpoint inhibitors (170).

## 6 Discussion

The overarching goal of this Review is to highlight the potential window of opportunity that the perioperative time period presents for identifying novel antitumor therapeutic strategies against pediatric solid tumors. As highlighted by the current understanding of the tumor immune microenvironment in these tumors, there are multiple and unique challenges that make harnessing the immune system against the tumor for therapy a challenge. Local control approaches with surgery and radiation induce systemic immune responses that can lead to a suppression of antitumor immunity. Thinking critically about how patients are medically managed through the time of surgery and radiation therapy may lead to novel breakthroughs and effective therapeutic strategies. An argument can be made for intrinsically changing the stimulus (i.e. surgery or radiation intensity) as a means to modulate the host-mediated responses to therapy. For example, utilizing minimally invasive surgical approaches such as laparoscopy or robotic access for tumor removal operations reduces the circulating levels of IL-6 post-operatively, indicating a reduced level of systemic inflammation. However, without significant new advances in surgical or radiation technology, this approach to limiting the negative systemic effects of a tumor removal operation or ablation with radiation is likely to have reached its near-term limit. Therefore, approaches to modulate the systemic host-mediated pro-tumorigenic response that is extrinsic to the actual surgical intervention are urgently needed to realize immediate benefit for patients.

## Author contributions

BC, MD and EV contributed to conception and design of the review. BC and EV wrote sections of the manuscript. All authors contributed to manuscript revision, read, and approved the submitted version.
